# Perinatal Oxidative Stress May Affect Fetal Ghrelin Levels in Humans

**DOI:** 10.1038/srep17881

**Published:** 2015-12-08

**Authors:** Zhong-Cheng Luo, Jean-François Bilodeau, Anne Monique Nuyt, William D. Fraser, Pierre Julien, Francois Audibert, Lin Xiao, Carole Garofalo, Emile Levy

**Affiliations:** 1 Ministry of Education-Shanghai Key Laboratory of Children’s Environmental Health, Xinhua Hospital, Shanghai Jiao-Tong University School of Medicine, Shanghai, China 200092; 2 Departments of Obstetrics and Gynecology, Pediatrics University of Montreal, Montreal, Canada H3T 1C5; 3 Departments of Obstetrics, Gynecology and Reproductive Medicine, Medicine University of Montreal, Montreal, Canada H3T 1C5; 4 Departments of Obstetrics and Gynecology, Nutrition University of Montreal, Montreal, Canada H3T 1C5; 5 Departments of Obstetrics and Gynecology, Sainte-Justine Hospital Research Center, University of Montreal, Montreal, Canada H3T 1C5; 6 Departments of Obstetrics and Gynecology, Molecular and Oncologic Endocrinology and Human Genomics Research Center, Quebec City, Canada G1V 4G2; 7 Departments of Obstetrics and Gynecology, University Hospital Research Center, Laval University, Quebec City, Canada G1V 4G2; 8 Department of Obstetrics and Gynecology, University of Sherbrooke, Sherbrooke, Canada J1H 5N4

## Abstract

In *vitro* cell model studies have shown that oxidative stress may affect beta-cell function. It is unknown whether oxidative stress may affect metabolic health in human fetuses/newborns. In a singleton pregnancy cohort (n = 248), we studied maternal (24–28 weeks gestation) and cord plasma biomarkers of oxidative stress [malondialdehyde (MDA), F2-isoprostanes] in relation to fetal metabolic health biomarkers including cord plasma glucose-to-insulin ratio (an indicator of insulin sensitivity), proinsulin-to-insulin ratio (an indicator of beta-cell function), insulin, IGF-I, IGF-II, leptin, adiponectin and ghrelin concentrations. Strong positive correlations were observed between maternal and cord plasma biomarkers of oxidative stress (r = 0.33 for MDA, r = 0.74 for total F2-isoprostanes, all p < 0.0001). Adjusting for gestational age at blood sampling, cord plasma ghrelin concentrations were consistently negatively correlated to oxidative stress biomarkers in maternal (r = −0.32, p < 0.0001 for MDA; r = −0.31, p < 0.0001 for F2-isoprostanes) or cord plasma (r = −0.13, p = 0.04 for MDA; r = −0.32, p < 0.0001 for F2-isoprostanes). Other fetal metabolic health biomarkers were not correlated to oxidative stress. Adjusting for maternal and pregnancy characteristics, similar associations were observed. Our study provides the first preliminary evidence suggesting that oxidative stress may affect fetal ghrelin levels in humans. The implications in developmental “programming” the vulnerability to metabolic syndrome related disorders remain to be elucidated.

Consistent evidence suggests that the perinatal period is a critical developmental time window in “programming” future risk of metabolic syndrome [obesity, impaired glucose tolerance, elevated blood pressure, high serum triglycerides and low serum high-density lipoprotein (HDL) levels] and related disorders (e.g. type 2 diabetes)^1^,^2^ How this vulnerability is developed during fetal life remains unclear. Oxidative stress - the loss of balance between pro-oxidation and anti-oxidation forces in the biological systems, has been associated with multiple perinatal adverse conditions including diabetes, preeclampsia, preterm birth and low birth weight that are predictive of an elevated risk of the metabolic syndrome in postnatal life^3^, and hence may be a common pathway in developmental metabolic programming^4^. Experimental studies in animal models and cell lines support the role of redox balance in modulating the expression of many genes^5^,^6^, and the beta-cell function could be a sensitive target to oxidative stress^7^,^8^,^9^. However, there is a lack of data on whether oxidative stress may affect metabolic health biomarkers in human fetuses/newborns. The present study sought to explore the hypothesis that perinatal oxidative stress may affect circulating levels of metabolic health biomarkers as related to fetal growth (insulin, IGF I and IGF II), insulin sensitivity, beta-cell function and energy regulation (leptin, adiponectin, ghrelin) in human fetuses/newborns. We studied leptin, adiponectin, ghrelin since they are important hormones in the regulation of energy balance and insulin sensitivity^10^. Interestingly, ghrelin is mainly secreted by the pancreas during fetal life, rather than the fundus of the stomach in adult humans^11^. It is unknown whether this “pancreatic” fetal hormone is related to perinatal oxidative stress.

## Methods

### Subjects and specimens

In a prospective pregnancy cohort study on fetal insulin sensitivity^12^, maternal and cord venous blood specimens were specifically collected for assays of biomarkers of oxidative stress for assessing its role in early life metabolic health. Briefly, a total of 339 healthy women (without pre-existing diabetes or other severe maternal illnesses) bearing a singleton fetus without malformation were recruited at 24–28 weeks gestation in Montreal (Sainte-Justine, Jewish General, and Saint Mary’s Hospitals) between August 2006 and December 2008. A total of 248 mother-infant pairs (73%) with maternal (24–28 weeks gestation) and cord plasma specimens available for assays of oxidative stress biomarkers constituted the final study cohort. There were 25 pregnancies complicated by gestational diabetes according to the 2003 American Diabetes Association’s 2-hour 75 g oral glucose tolerance test (OGTT) diagnostic criteria^13^, fourteen pregnancies complicated by gestational hypertension (including 3 cases of preeclampsia), and 11 by preterm deliveries (all mild preterm, 33–36 weeks). They were included since their exclusions did not affect all results. The characteristics of the study cohort have been described previously^12^.

Maternal venous blood specimens were collected at 24–28 weeks gestation, and cord venous blood specimens were collected *immediately* after delivery of the baby but before delivery of the placenta. A tube of EDTA blood sample was *specifically* collected for assays of oxidative stress biomarkers by adding 0.1% butylated hydroxytoluene to prevent oxidation after specimen collection. All blood specimens were kept on ice and centrifuged within 30 minutes after collection. Plasma specimens were stored in multiple aliquots at −80 °C until biochemical assays.

### Ethics statement

The study was approved by the Research Ethics Committee of Sainte-Justine Hospital Research Center, University of Montreal, and adhered to the tenets and guidelines of the Declaration of Helsinki. Written informed consent was obtained from all participants.

### Biochemical assays

Plasma total F2-isoprostanes including 7 isomers (8-iso-PGF2α, 15(R)-PGF2α, 8-iso-15(R)-PGF2α, iPF2α-IV, iPF2α-VI, 5-iPF2α-VI, 5-8,12-iso-iPF2α-VI) (pg/ml) were measured by high-performance liquid chromatography tandem mass spectrometry (HPLC-MS/MS) using a column packed with core-shell particles^14^. The intra- and inter-assay coefficients of variation (CVs) were in the range of 2.6% to 8.2%. Plasma malondialdehyde (MDA) was measured by HPLC with fluorescence detectionx^14^. The intra- and inter-assay CVs were in the range of 3.6% to 6.8%.

Plasma unacylated ghrelin was measured by a human unacylated ghrelin immunoassay kit (SPI-BIO, Bertin). The intra- and inter-assay coefficients of variation (CVs) were in the range of 2.8% to 9.2%. We chose to measure unacylated ghrelin since it accounts for about 99% of total ghrelin, while acylated ghrelin accounts for only about 1% of total ghrelin in cord blood^15^. Also, for accurate measurement of acylated ghrelin, specimens need special treatment to prevent the degradation of acylated ghrelin, but this was not done during specimen collection and processing in the study. Without timely anti-deacylation treatment, acylated ghrelin would be rapidly converted to unacylated ghrelin in blood samples^16^. Therefore, the measurement of plasma unacylated ghrelin is effectively the measurement of total ghrelin in the present study. For simplicity, we used the term ghrelin in lieu of unacylated ghrelin in results presentation.

Data on maternal and cord plasma concentrations of leptin and adiponectin, insulin-like growth factor I (IGF-I), IGF-II and surrogate indicators of fetal insulin sensitivity (cord plasma glucose-to-insulin ratio) and beta cell function (proinsulin-to-insulin ratio) are available in the study cohort^12^,^17^,^18^. The intra- and inter-assay CVs in the assays of plasma glucose, insulin, proinsulin, IGF-I, IGF-II, leptin and adiponectin were in the range of 2.0% to 10.4%.

All assays were blinded to the labs. The lab staff had no information on clinical characteristics of study subjects.

### Oxidative stress biomarkers

Maternal and fetal oxidative stress levels were represented by plasma total F2-isoprostanes and MDA concentrations. F2-isoprotanes, the products of lipid peroxidation, are reliable biomarkers of *in vivo* oxidative stress^19^. MDA, another product of lipid peroxidation, was used for evaluating the consistency of associations between oxidative stress and fetal metabolic health biomarkers. Many previous studies used enzyme-linked immuno-assays to measure only one specific isomer of F2-isoprostaness - the 8-iso-PGF2α which accounts for only a small fraction of total F2-isoprostanes^14^, as an oxidative stress biomarker. The use of our recently developed technique for measuring F2-isoprostanes by HPLC-MS/MS allows the simultaneous measurements of seven isomers. In addition to total F2-isoprostanes, we explored the associations of specific F2-isoprostane isomers with fetal metabolic health biomarkers.

### Fetal metabolic health biomarkers

Fetal metabolic health biomarkers included cord plasma glucose-to-insulin ratio (a surrogate indicator of fetal insulin sensitivity)^20^ proinsulin-to-insulin ratio (a surrogate indicator of beta-cell function^21^), insulin, IGF-I and IGF-II (key fetal growth hormones)^17^, leptin, adiponectin and ghrelin concentrations. Leptin, adiponectin and ghrelin are important hormones in regulating insulin sensitivity and energy balance^22^,^23^. We hypothesized that perinatal oxidative stress may “program” long-term metabolic risk through affecting one or more of these fetal growth and metabolic health biomarkers. Birth weight (z score, according to the Canadian sex- and gestational age-specific birth weight standards^24^) was also examined because oxidative stress has been associated with impaired fetal growth in some studies^25^,^26^.

### Statistical analysis

Median (inter-quartile range) and Mean±SD (standard deviation) are presented for plasma ghrelin, MDA and F2-isoprostanes concentrations. Biomarker data (positively skewed crude data distribution) were log-transformed in partial correlation analyses adjusting for gestational age at blood sampling (maternal or cord blood) and glucose concentration (an important indicator of metabolic status at the time of blood sampling), and in t test or analysis of variance for differences between groups where appropriate. Generalized linear models were used to assess the associations of oxidative stress with cord plasma metabolic health biomarkers in log-transformed data adjusting for maternal and pregnancy characteristics. The effects were expressed in percentage change [for a dependent variable (y) in log scale, the regression coefficient (β) effectively represents the proportion of change in the original scale [log y_1_ – log y_0_ = β, then log (y_1_/y_0_) = β, thus y_1_/y_0_ = e^β^, and the percentage change is (e^β^ -1)*100%]. The co-variables considered for inclusion in the adjusted models were maternal race (White, others), age (<35, ≥35 years), parity (primiparous: yes/no), pre-pregnancy BMI (kg/m^2^), smoking (yes/no), alcohol use (yes/no), gestational diabetes (yes/no), gestational hypertension (yes/no), infant sex (boy, girl), gestational age (week), ponderal index (birth weight/length^3^, kg/m^3^), mode of delivery (cesarean, vaginal) and cord plasma glucose concentration (SD score). Co-variables with p values <0.20 were retained in the parsimonious final adjusted models. Birth weight was not included in the adjusted models since it was highly correlated with ponderal index, a surrogate indicator of fetal adiposity associated with insulin sensitivity^12^,^27^. Data management and analyses were conducted using Statistical Analysis System (SAS), Version 9.2 (SAS Institute, Cary, North Carolina). P values <0.003 were considered statistically significant, accounting for 16 primary comparisons of interest (8 cord plasma biomarkers as the primary outcomes, 2 cord plasma oxidative stress biomarkers as the primary exposures, total number of primary comparisons = 8*2 = 16, adjusted p value cutoff = 0.05/16 = 0.003). The study had a power of 99% to detect an absolute correlation efficient of 0.3 or greater, taking into account of multiple tests.

## Results

Comparing plasma concentrations in cord vs. maternal blood, MDA (median: 332.2 vs. 168.2 pmol/ml) and total F2-isoprostanes (median: 3232.1 vs.2320.9 pg/ml) were all significantly higher (Table 1). Plasma concentrations were higher in cord versus maternal blood in each of the seven F2-isoprostane isomers. Ghrelin concentrations were also much higher in cord vs. maternal plasma. Descriptive statistics on other biomarkers (glucose-to-insulin ratio, proinsulin-to-insulin ratio, IGF-I, IGF-II, leptin, adiponectin) in this cohort have been reported in previous works^12^,^17^,^18^, and thus are not presented here.

Gestational age at birth was positively correlated to cord plasma leptin (crude r = 0.22, p = 0.0007) and adiponectin (r = 0.19, p = 0.0025) concentrations, but negatively correlated to cord plasma IGF-I (r = −0.22, p = 0.0001) concentrations. There were no significant correlations between gestational age and cord plasma MDA (r = −0.10, p = 0.12), F2-isoprostanes (r = −0.06, p = 0.36), ghrelin (r = 0.10, p = 0.12), insulin (r = −0.11, p = 0.09), proinsulin (r = −0.12, p = 0.05) and IGF-II (r = 0.09, p = 0.17) concentrations.

**Figure 1:**
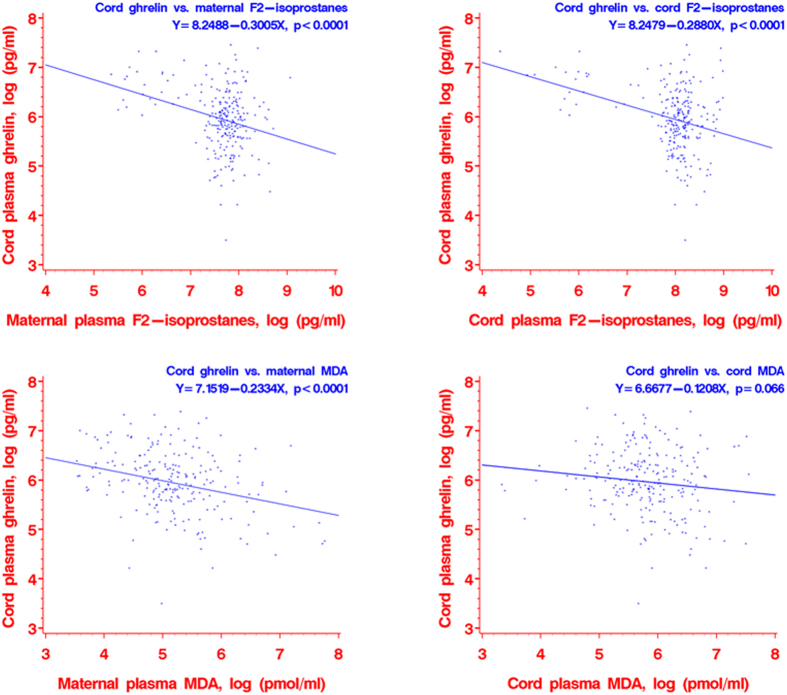
Scatter plots of cord plasma ghrelin concentrations in relation to oxidative stress biomarkers [F2-isoprostanes and MDA (malondialdehyde)] in maternal and cord plasma. All data presented are in log scale.

Scatter plots revealed negative relationships between maternal or cord plasma F2-isoprostanes and cord plasma ghrelin concentrations (tests for linear trends in log transformed data, all p < 0.0001) (Fig. 1), and between maternal plasma MDA and cord plasma ghrelin concentrations (p < 0.0001). There was also a negative relationship between cord plasma MDA and ghrelin concentrations, but the association was not statistically significant (p = 0.066).

Adjusting for gestational age at maternal and cord blood sampling and glucose concentration, significant positive correlations were observed between maternal and cord plasma concentrations in ghrelin (partial r = 0.33, p < 0.0001) and biomarkers of oxidative stress (partial r = 0.33 for MDA, r = 0.74 for F2-isoprostanes, all p < 0.0001) (Table 2). Consistent negative correlations were observed between cord plasma ghrelin levels and indices of oxidative stress in both maternal (partial r = −0.32, p < 0.0001 for MDA; r = −0.31, p < 0.0001 for total F2-isoprostanes) and cord plasma (partial r = −0.13, p = 0.04 for MDA; r = −0.32, p < 0.0001 for total F2-isoprostanes). There were no significant correlations in maternal or cord plasma concentrations of MDA or total F2-isoprostanes with cord plasma concentrations of insulin, leptin, adiponectin, IGF-I or IGF-II. Similarly, there were no significant correlations in oxidative stress biomarkers with cord plasma glucose-to-insulin and proinsulin-to-insulin ratios (Table 2), or with cord plasma proinsulin concentrations (data not shown).

Exploratory analyses showed that cord plasma ghrelin concentrations were negatively correlated with four specific F2-isoprostane isomers (partial r = −0.34 for 8-iso-PGF2α, p < 0.0001; r = −0.31 for 15(R)-PGF2α, p < 0.0001; r = −0.34 for 8-iso-15(R)-PGF2α, p < 0.0001; r = −0.17 for iPF2α-IV, p = 0.007), but no significant correlations with the other three F2-isoprostane isomers (r = −0.09 for iPF2α-VI, p = 0.15; r = −0.03 for 5-iPF2α-VI, p = 0.63; r = 0.05 for 5–8,12-iso-iPF2α-VI, p = 0.49). There were no significant correlations between all specific F2-isoprostane isomers and cord plasma leptin or adiponectin concentrations (all p > 0.1), except for that cord plasma concentrations of 5-iPF2α-VI and adiponectin were weakly correlated (r = 0.16, p = 0.02).

Adjusting for maternal plasma ghrelin concentration, pre-pregnancy BMI, infant sex, gestational age, ponderal index and mode of delivery (other co-variables were excluded at p > 0.2 in multivariate generalized regression models), significant negative associations remained in maternal or cord plasma F2-isoprostanes and maternal plasma MDA concentrations with cord plasma ghrelin concentrations (Table 3). For each log unit increase in plasma concentration, cord plasma ghrelin decreased by 14.2% for maternal plasma MDA (adjusted p < 0.001), by 10.7% for maternal plasma total F2-isoprostanes (adjusted p = 0.039), and by 14.0% for cord plasma total F2-isoprostanes (adjusted p = 0.002), respectively. The association between cord plasma MDA and ghrelin became not statistically significant (p = 0.206) after the multivariate adjustment, but the point estimate also showed a negative association. There was a strong influence of maternal ghrelin on fetal circulating ghrelin concentration: each log (pg/ml) unit increase in maternal ghrelin was associated with a 68% increase in cord plasma ghrelin (p < 0.001).

Cord plasma ghrelin concentration was negatively correlated with birth weight z score (according to the Canadian sex and gestational age-specific fetal growth standards (21)) (r = −0.15, p = 0.02). Large-for-gestational-age (LGA > 90^th^ percentile, n = 27) infants had lower ghrelin concentrations than AGA infants (median: 306.4 vs. 397.2 pg/ml, p = 0.03). However, cord plasma ghrelin concentrations were not significantly different comparing birth weight small-for-gestational-age (SGA < 10^th^ percentile, n = 14) vs. appropriate-for gestational age (AGA) infants (median: 443.0 vs. 397.2 pg/ml, p = 0.55).

There were no significant correlations between birth weight (z score) and oxidative stress biomarkers (MDA or F2-isoprostanes) (all p > 0.05, data not shown). Cord plasma concentrations of MDA, total F2-isoprostanes or specific F2-isoprostane isomers were similar comparing SGA vs. AGA newborns, except for that SGA infants had lower 5-iPF2α-VI concentrations (median: 228.3 vs. 262.6 pg/ml, p = 0.01). There were no significant differences in cord plasma biomarkers of oxidative stress in LGA vs. AGA infants.

There were no significant differences in maternal and cord plasma concentrations of ghrelin or biomarkers of oxidative stress comparing gestational diabetic (n = 25) versus non-diabetic pregnancies, or gestational hypertensive (n = 14) versus non-hypertensive pregnancies, or preterm (n = 11) vs. term births (all p > 0.05, data not shown). Excluding these patients from the analyses, the results were similar. There were no significant differences in cord plasma biomarkers of oxidative stress in caesarean-section (n = 70) vs. vaginal deliveries, or between boys and girls (all p > 0.05, data not shown).

## Discussion

### Main findings

To our knowledge, this is the first study demonstrating a consistent negative correlation in perinatal (maternal or fetal) oxidative stress with circulating ghrelin levels in human fetuses/newborns. Also, this is the largest pregnancy cohort demonstrating that maternal and fetal oxidative stress levels are strongly positively correlated.

### Data interpretation in comparisons with previous studies

The developmental programming hypothesis is well recognized^2^, but data on perinatal biomarkers that may be related to the mechanisms of metabolic programming remain scanty in humans^28^. The present study provides important evidence suggesting that perinatal oxidative stress does not affect most fetal metabolic health indicators as measured at birth in a relatively low-risk pregnancy cohort (without pre-gestational serious chronic illnesses such as diabetes or hypertension). However, oxidative stress was associated with decreased fetal ghrelin levels. In addition, there is a strong influence of maternal ghrelin levels on fetal ghrelin levels.

Ghrelin is an important hormone in regulating food intake and energy balance, and is primarily secreted by the fundus of the stomach in adult humans and rodents, but is primarily secreted by the pancreas during the perinatal period^11^. Ghrelin circulates in two forms: unacylated and acylated. Acylated ghrelin is considered the biologically active form with respect to its growth hormone secretion stimulation and orexigenic effects through binding to growth hormone secretagogue receptor^29^. However, unacylated ghrelin is not biologically inactive, and may counter the metabolic effects of acylated ghrelin^30^. Unacylated ghrelin in the predominant form in fetal circulation^11^, but little is known about its biological relevance. A recent intervention study demonstrated a negative association between oxidative stress and circulating ghrelin concentrations in adults^31^. There is an absence of data on perinatal oxidative stress in relation to ghrelin levels in fetuses/newborns. We observed a consistent negative correlation between maternal or fetal oxidative stress and fetal ghrelin levels. On the other hand, it has been proposed that both acylated and unacylated ghrelin may have a protective effect against oxidative stress in cell and animal model studies^32^,^33^. There is a possibility of reverse causality that lower ghrelin levels might be a cause of higher oxidative stress levels in the newborns. However, the consistent negative correlations of both maternal and cord blood oxidative stress biomarkers with cord ghrelin levels are reassuring, since it is unlikely that fetal ghrelin would cause oxidative stress in the mothers. The implications of fetal ghrelin concentration for postnatal metabolic health are unknown. Considering the plurality of biological effects of ghrelin, it is possible that the oxidative stress associated changes in fetal ghrelin levels may be related to a developmental metabolic programming effect on long-term susceptibility to obesity and metabolic syndrome in postnatal life.

Interestingly, we observed that the associations between F2-isoprostanes and fetal circulating ghrelin levels appear to be restricted to certain isomers (8-iso-PGF2α, 15(R)-PGF2α, 8-iso-15(R)-PGF2α, and iPF2α-IV). This finding requires confirmation from other independent cohort studies. F2-isoprostane isomers are biological active molecules. The 8-iso-PGF2α is the most studied F2-isoprostane isomer which has been associated with potent vascular constrictive effects, while research on the biological effects of other isomers has been limited, and isomer-specific biological effects may exist^34^. Much remains to be known about the biological effects of various F2-isoprostane isomers. There is an absence of data on the physiological relevance of various F2-isoprostane isomers in metabolic health. Our preliminary data indicate that certain F2-isoprostanes may be related to ghrelin synthesis or secretion during fetal development.

Apart from ghrelin, we did not observe any significant association between oxidative stress and other fetal metabolic health biomarkers including leptin, adiponectin, glucose-to-insulin ratio, proinsulin-to-insulin ratio, insulin, IGF-I and IGF-II. Also, we could not confirm an association between oxidative stress and poor fetal growth. Caution is warranted in data interpretation since the number of SGA infants is small (n = 14) in the study cohort. It should be noted that there is a tendency of under-reporting null association in the literature^35^. It has been recognized that fetal metabolic programming may occur within normal birth weight ranges^2^. It appears that in this relatively low risk pregnancy cohort (free of major pre-gestational illnesses), there was no significant impact of perinatal oxidative stress on fetal growth and most fetal metabolic health biomarkers. Preterm delivery has been associated with elevated insulin levels at birth and early childhood in a recent large prospective cohort study^36^. We could not detect any significant differences in cord blood oxidative stress and metabolic health biomarkers in preterm vs term births, but caution is warranted in data interpretation since the number of preterm infants is small.

### Strengths and limitations

Main study strengths are the largest pregnancy cohort on maternal and fetal oxidative stress, and objective biomarker measurements (assay labs’ blindness to patients’ clinical characteristics). Main study limitation is that we only have data on total ghrelin, but could not detect different forms of ghrelin. However, it is known that concentrations of acylated and unacylated ghrelin are highly correlated, and unacylated ghrelin accounts for about 99% of total ghrelin in cord blood^15^. The study is observational in nature, and we could speculate, but not affirm that the observed associations are causal.

## Conclusions

Oxidative stress was negatively correlated with circulating ghrelin concentrations, but not associated with insulin, leptin, adiponectin, IGF-I and IGF-II concentrations, or glucose-to-insulin and proinsulin-to-insulin ratios in human fetuses/newborns. The implications in developmental metabolic programming remain to be understood. The observations invite the hypothesis that oxidative stress in early life may affect fetal ghrelin levels to “program” the vulnerability to metabolic syndrome and related disorders in adulthood.

## Additional Information

**How to cite this article**: Luo, Z.-C. *et al.* Perinatal Oxidative Stress May Affect Fetal Ghrelin Levels in Humans. *Sci. Rep.* **5**, 17881; doi: 10.1038/srep17881 (2015).

## Figures and Tables

**Table 1:** Maternal and cord plasma concentrations of oxidative stress biomarkers (MDA, F_2_-Isoprostanes) and ghrelin in mother-newborn pairs (N = 248).

	Maternal plasma (24–28 weeks gestation)		Cord plasma		
	Median (IQR)	Mean±SD	Median (IQR)	Mean±SD	P^ξ^
MDA, pmol/ml	168.2 (106.6, 266.5)	244.6 ± 311.8	332.2 (238.1, 514.4)	413.3 ± 298.4	< 0.0001
F_2_-Isoprostanes, pg/ml					
8-iso-PGF_2α_	129.1 (92.5, 186.3)	146.8 ± 108.6	189.1 (133.9, 283.0)	240.9 ± 223.8	< 0.0001
15(R)-PGF_2α_	430.7 (354.3, 540.8)	462.5 ± 236.3	531.9 (431.5, 738.7)	612.1 ± 378.6	< 0.0001
8-iso-15(R)-PGF_2α_	127.8 (92.8, 126.0)	128.9 ± 66.7	202.4 (156.9, 281.5)	218.8 ± 127.7	< 0.0001
iPF_2α_-IV	68.7 (60.0, 80.5)	70.6 ± 30.1	76.1 (66.1, 92.9)	82.6 ± 42.0	< 0.0001
iPF_2α_-VI	297.5 (249.2, 398.1)	390.6 ± 591.4	466.2 (374.4, 658.2)	598.8 ± 637.6	< 0.0001
5-iPF_2α_-VI	188.4 (161.5, 212.3)	212.4 ± 140.5	261.1 (223.1, 306.4)	306.0 ± 201.1	< 0.0001
( ± )5-8,12-iso-iPF_2α_-VI	966.5 (808.3, 1304.9)	1123.0 ± 587.5	1384.7 (1134.4, 1691.6)	1517.2 ± 634.7	< 0.0001
*Total F*_*2*_*-Isoprostanes*	2320.9 (1884.6, 2802.8)8)	2370.0 ± 1084.3	3232.1 (2720.7,3844.3 )	3358.3 ± 1465.5	< 0.0001
Ghrelin, pg/ml	130.0 (83.7, 178.5)	153.6 ± 119.7	392.8 (268.9, 570.1)	465.2 ± 297.5	< 0.0001

MDA = malondialdehyde; IQR = inter-quartile range (25^th^, 75^th^); SD = standard deviation.

^ξ^P value in paired t tests for differences comparing concentrations in cord plasma versus maternal plasma in log-transformed data.

**Table 2:** Partial correlation coefficients (n = 248 mother-newborn pairs) between oxidative stress and fetal (cord plasma) metabolic health biomarkers.^ξ^

	Maternal plasma (24–28 weeks)		Cord plasma		
	MDA	F_2_-isoP	MDA	F_2_-isoP	Ghrelin
Mother plasma					
F_2_-isoP	**0.31**^**τ**^				
Ghrelin	**−0.23‡**	**−0.39**^**τ**^	−0.07	**−0.33**^**τ**^	**0.51**^**τ**^
Cord plasma					
MDA	**0.33**^**τ**^	0.09			
F_2_−IsoP	0.20†	**0.74**^**τ**^	0.11		
Ghrelin	**−0.31**^**τ**^	**−0.31**^**τ**^	−0.13*	**−0.32**^**τ**^	
Leptin	−0.05	−0.18†	−0.02	−0.06	0.05
Adiponectin	−0.05	−0.17†	0.01	−0.03	0.06
Insulin	0.04	−0.01	0.04	0.03	−0.02
Glucose/insulin	−0.02	−0.06	−0.01	−0.09	0.05
Proinsulin/insulin	−0.03	−0.03	−0.07	0.06	−0.06
IGF-1	0.01	0.03	−0.06	0.02	−0.15*
IGF-II	0.01	−0.07	−0.04	−0.04	−0.04
Birth weight (z)	0.05	0.04	−0.09	−0.03	-0.16†

^ξ^Partial correlations adjusting for gestational age at blood sampling (maternal and cord blood) and glucose concentration (an important indicator of metabolic status at the time of blood sampling); data were log-transformed for biomarkers with skewed crude data distribution before the correlation analyses. Correlation coefficients in bold remained significant after accounting for multiple tests at p < 0.001.

^*^p < 0.05, †p < 0.01, ‡p < 0.001, τ p < 0.0001. MDA = malondialdehyde; F_2_-Isop = total F_2_-isoprostanes.

**Table 3:** Adjusted percentage change (95% CI)ξ in cord plasma ghrelin concentration in relation to maternal plasma ghrelin, maternal and cord plasma MDA and F2-isoprostanes concentrations.

Per log unit increase in concentration of:	Adjusted % change (95% CI) in cord plasma plasma ghrelin concentration ξ	P
Maternal plasma (24–28 weeks)		
MDA	−14.2 (−20.9, −7.0)	< 0.001
Total F_2_-isoprostanes	−10.7 (−19.9, −0.6)	0.039
Ghrelin	68.1 (50.4, 87.9)	< 0.001
Cord plasma		
MDA	−6.9 (−16.8, 4.1)	0.206
Total F_2_-isoprostanes	−14.0 (−21.8, −5.3)	0.002

^ξ^Co-variables considered for inclusion in the adjusted models were maternal race, age, parity, pre-pregnancy body mass index, smoking, alcohol use, gestational diabetes, gestational hypertension, infant sex, gestational age, ponderal index, mode of delivery and cord plasma glucose concentration; only co-variables with p values < 0.20 were retained in the final adjusted models (*pre-pregnancy BMI, infant sex, gestational age, ponderal index and mode of delivery*). The models for the effects of MDA and F_2_-isoprostanes on cord ghrelin concentration were further adjusted for maternal ghrelin concentration. *Separate* models were fitted for maternal and cord blood oxidative stress biomarkers (MDA, total F2-isoprostanes) as they were strongly correlated. Model’s R squares are in the range of 0.34 to 0.39. The adjusted % change was calculated from the regression coefficient of the dependent variable (y) in log scale per log unit increase in the independent variable (x), because the regression coefficient (β) represents the proportion of change in y in the original scale (ghrelin concentration): log y_1_ – log y_0_ = β, then log (y_1_/y_0_) = β, thus y_1_/y_0_ = e^β^, and the percentage change is (e^β^ -1)*100%.

MDA = malondialdehyde; CI = confidence interval.
